# Cyclosporin A-Mediated Activation of Endogenous Neural Precursor Cells Promotes Cognitive Recovery in a Mouse Model of Stroke

**DOI:** 10.3389/fnagi.2018.00093

**Published:** 2018-04-24

**Authors:** Labeeba Nusrat, Jessica M. Livingston-Thomas, Vaakiny Raguthevan, Kelsey Adams, Ilan Vonderwalde, Dale Corbett, Cindi M. Morshead

**Affiliations:** ^1^Department of Surgery, University of Toronto, Toronto, ON, Canada; ^2^Institute of Medical Sciences, University of Toronto, Toronto, ON, Canada; ^3^Institute of Biomaterials and Biomedical Engineering, University of Toronto, Toronto, ON, Canada; ^4^Department of Cellular & Molecular Medicine, University of Ottawa, Ottawa, ON, Canada; ^5^Canadian Partnership for Stroke Recovery, Ottawa, ON, Canada

**Keywords:** stroke, cognitive dysfunction, neural precursor cells, immunosuppression, neural precursor activation, cognitive recovery, endogenous repair

## Abstract

Cognitive dysfunction following stroke significantly impacts quality of life and functional independance; yet, despite the prevalence and negative impact of cognitive deficits, post-stroke interventions almost exclusively target motor impairments. As a result, current treatment options are limited in their ability to promote post-stroke cognitive recovery. Cyclosporin A (CsA) has been previously shown to improve post-stroke functional recovery of sensorimotor deficits. Interestingly, CsA is a commonly used immunosuppressant and also acts directly on endogenous neural precursor cells (NPCs) in the neurogenic regions of the brain (the periventricular region and the dentate gyrus). The immunosuppressive and NPC activation effects are mediated by calcineurin-dependent and calcineurin-independent pathways, respectively. To develop a cognitive stroke model, focal bilateral lesions were induced in the medial prefrontal cortex (mPFC) of adult mice using endothelin-1. First, we characterized this stroke model in the acute and chronic phase, using problem-solving and memory-based cognitive tests. mPFC stroke resulted in early and persistent deficits in short-term memory, problem-solving and behavioral flexibility, without affecting anxiety. Second, we investigated the effects of acute and chronic CsA treatment on NPC activation, neuroprotection, and tissue damage. Acute CsA administration post-stroke increased the size of the NPC pool. There was no effect on neurodegeneration or lesion volume. Lastly, we looked at the effects of chronic CsA treatment on cognitive recovery. Long-term CsA administration promoted NPC migration toward the lesion site and rescued cognitive deficits to control levels. This study demonstrates that CsA treatment activates the NPC population, promotes migration of NPCs to the site of injury, and leads to improved cognitive recovery following long-term treatment.

## Introduction

Stroke is a leading cause of death and chronic disability. Prevalence of post-stroke cognitive dysfunction can be up to 80% of stroke survivors (Sun et al., 2014), including impairments in learning and memory and deficits in executive functions (Zinn et al., 2007; Hofmann et al., 2012; Douiri et al., 2013). Such impairments can persist for years (Poulin et al., 2012) and are associated with higher rates of long-term post-stroke disability (Cumming et al., 2013; Mullick et al., 2015), impacting the quality of life of survivors. Areas of the prefrontal cortex, such as anterior cingulate and dorsolateral prefrontal cortices, are responsible for regulating executive functioning which is commonly affected after stroke (Kumral et al., 2002; Pohjasvaara et al., 2002; Poulin et al., 2012). Executive functions include an array of complex behaviors that govern self-regulatory actions and goal-management such as problem solving, cognitive flexibility, decision making and working memory (Zinn et al., 2007; Hofmann et al., 2012; Cordova et al., 2014) Despite the profound impact of cognitive deficits after stroke, therapeutic strategies to promote long-term cognitive recovery have attracted relatively little attention (Zinn et al., 2007; Cumming et al., 2013). Currently, cognitive rehabilitation which targets spared skills to develop compensatory strategies is the only treatment option for stroke survivors, and has limited efficacy (Adams et al., 2007; Poulin et al., 2012). Thus, it is timely to consider the development of strategies to enhance neural repair for the treatment of post-stroke cognitive deficits.

The adult mammalian brain contains neural stem cells that possess the ability to self-renew and generate progeny that differentiate into neural phenotypes, making them promising therapeutic targets to promote neural regeneration and plasticity. Stem cells and their progeny (together termed neural precursor cells, NPCs) exist in the subependyma (SE) lining the walls of the lateral ventricles, and the subgranular zone of dentate gyrus, where they contribute to ongoing neurogenesis in the olfactory bulb and hippocampus, respectively (Doetsch, 2003). A number of factors have been shown to activate endogenous NPCs. For example, brain injury activates endogenous NPCs, leading to an expansion of the size of the NPC pool, migration, and neurogenesis (Parent et al., 2002; Jin et al., 2003; Ahmed et al., 2014) Administration of growth factors and small molecules can similarly promote NPC expansion, migration and differentiation (Kolb et al., 2007; Hunt et al., 2010; Dadwal et al., 2015). We have previously demonstrated that the commonly used immunosuppressant, Cyclosporin A (CsA), has direct pro-survival effects on NPCs both *in vitro* and *in vivo*, thereby expanding the size of the NPC pool (Hunt et al., 2010; Chow and Morshead, 2016). Interestingly, while the immunosuppressant effects of CsA are mediated by a calcineurin-dependent pathway, with downstream effects resulting in T-cell regulation, the NPC pro-survival effects of CsA result from activation of a calcineurin-independent pathway (Sachewsky et al., 2014), which inhibits mitochondrial pore formation and effectively blocks apoptotic pathways. In models of sensorimotor stroke, CsA administration leads to NPC pool expansion, tissue regeneration, and motor recovery (Erlandsson et al., 2011; Sachewsky et al., 2014). Thus, CsA shows therapeutic promise, and herein we propose to study its efficacy for the treatment of cognitive impairments after stroke.

To investigate the potential of this endogenous repair strategy to promote cognitive recovery following stroke, we first established a reproducible cognitive stroke model in mice. Based on a similar model in rats (Livingston-Thomas et al., 2015), bilateral focal lesions were produced using endothelin-1 (ET-1) in the medial prefrontal cortex, which is analogous to the human cingulate and dorsolateral prefrontal cortices (Seamans et al., 2008; Cordova et al., 2014). Stroke-injured mice displayed significant short- and long-term cognitive deficits, and an expansion in the size of the NPC pool similar to that observed following ET-1-induced sensorimotor stroke (Sachewsky et al., 2014). Chronic CsA treatment promoted migration of NPCs to the site of the lesion, and rescued cognitive performance to control levels following stroke, demonstrating the therapeutic potential of this endogenous NPC activation strategy to enhance cognitive recovery following stroke.

## Materials and Methods

### Animals

This study was carried out in accordance with the recommendations of guidelines of the University of Toronto Animal Care Committee and the Canadian Council of Animal Care. The protocol was approved by the University of Toronto Animal Care Committee. Sixty adult male C57BL/6 mice (6–8 weeks of age; 20–25 g; Charles River) were single housed on a 12-h light/dark cycle with food and water *ad libitum*. For each experiment, specific *n*-numbers are presented in the figure captions. Mice were acclimated to the facility for 1 week prior to the experiment. Mice were randomly assigned to the uninjured Control, Stroke-alone, or Stroke+CsA group.

### Stroke and CsA Administration

To induce mPFC stroke, mice received stereotaxic microinjections of ET-1 (0.76 μl; 800 picomolar; Calbiochem) at two bilateral injection sites (for a total of four injections): (1) anteroposterior (AP) +2.2 mm from Bregma; mediolateral (ML) ±0.4 mm from midline; dorsoventral (DV) −2.4 mm from skull surface, and (2) AP +1.5 mm; ML ±0.4 mm; DV 2.6 mm. Immediately following injections, mini osmotic pumps (Alzet, Cupertino, CA, USA) filled with CsA (15 mg/kg/day; dissolved in 65% ethanol and 35% cremaphor) were implanted subcutaneously and replaced as required based on the length of the experiment. Sensorimotor stroke was induced as described previously (Sachewsky et al., 2014).

### Neurosphere Assay

Animals were anesthetized with isoflurane, quickly decapitated, and the brain was removed. The adult SE was dissected and cultured as previously described. Briefly, tissue was collected and enzymatically dissociated, triturated into a single cell suspension and plated at clonal density (10 cells/μl) in serum free media (SFM) supplemented with EGF (20 ng/ml), FGF (10 ng/ml) and heparin (7.35 ng/ml; all from Sigma; Coles-Takabe et al., 2008). The number of neurospheres was counted following 7 days in culture. Neurospheres originate from a single stem cell; hence, the number of neurospheres reflects the size of the NPC pool (Hunt et al., 2010). The assay was performed at 7 and 60 days following stroke. For cortical neurosphere assays, tissue was dissected from the lesioned bilateral mPFC region and plated as described (Erlandsson et al., 2011).

### Behavioral Testing

All behavioral testing took place after stroke surgery, with the exception of the adhesive removal test which included baseline testing prior to injury. Prior to testing, all animals were acclimated to the testing room for at least 10 min.

#### Adhesive Removal Test

To screen for sensorimotor deficits, the adhesive removal test was performed as previously described (Bouet et al., 2009). A 0.3 × 0.4 cm piece of tape was placed on the center of each forepaw, and the latency to contact and then remove the tape was measured. A functional score was calculated as ([latency to remove] − [latency to contact]). The task consisted of 7 days of training to ensure performance under 1 min, and 2 days of testing on day 4 and day 5 after stroke. Post-stroke performance was averaged from the 2 days of testing for analysis.

#### Puzzle Box Task

The Puzzle Box (PB) task is used to assess cognitive functions including problem-solving, and short- and long-term memory (Ben Abdallah et al., 2011). Mice were placed into a brightly-lit start zone of a rectangular box, and were required to travel to a dark, enriched goal zone. The test was performed as three trials per day (maximum 300 s) with a 2 min inter-trial interval, for three consecutive days. The test was performed at three time points throughout the experiment: post stroke days 4–6, 22–24 and 45–47. Trials were filmed from above using Viewer software (Bioserve, Bonn, Germany).

On Trial 1, mice were allowed easy access to the goal zone through an open door and clear underpass. Trials 2 and 3 required access through the underpass only, and the doorway blocked. The following day, the same paradigm was repeated (Trial 4; long term memory), following which accessibility was made more difficult by blocking the underpass with bedding, requiring which mice to remove it in order to enter the goal zone (Trial 5; problem solving). That paradigm was then repeated in Trial 6 (short-term memory). On the last day, the paradigm is repeated once more (Trial 7; long-term memory), following which the underpass is blocked with a cardboard plug that must be removed (Trial 8; problem solving). Lastly, that paradigm is repeated (Trial 9; short-term memory).

#### Morris Water Maze

The morris water maze (MWM) evaluates spatial learning, long-term memory, and behavioral flexibility. The test takes place over three phases: acquisition (5 days), probe (1 day) and reversal (4 days). During the acquisition phase, animals must locate a static hidden platform located in a circular pool of water. Mice were given four trials/day, each from a pseudorandomly assigned quadrant, with a maximum trial time of 60 s. Once the platform was located, mice were returned to their home cage for a 15 min inter-trial interval. The latency to locate the platform was recorded as the average trial time over 5 days. Twenty-four hours following the last acquisition trial, a probe test was performed. The platform was removed, mice were placed into the pool at a randomly assigned start location, and allowed to search for the platform for 60 s. The proportion of time spent searching in the maze quadrant that previously contained the platform was recorded. Twenty-four hours later the reversal phase began, for which the platform was replaced in the quadrant opposite from the original location, and trials were run as described in the acquisition phase. Mice were tested from days 35–44 post-stroke.

#### Open Field Task

The open field task was used to assess anxiety-related behavior. Mice were placed into a 60 × 60 cm chamber conceptually divided into 16 equal squares. The central eight squares were defined as the central zone, and the remaining squares were considered the edge. Animals were allowed to explore the environment for 10 min. Locomotor activity in the central and edge zones were evaluated on day 7 and 48 post-stroke.

### Tissue Preparation and Histology

All animals were deeply anesthetized with tribromoethanol (250 mg/kg; Sigma-Aldrich) and transcardially perfused with 0.01 M phosphate buffered saline (PBS), followed by 4% paraformaldehyde. Brains were collected and stored in 4% paraformaldehyde overnight, then transferred to 20% sucrose solution until saturated. Coronal brain sections (20 μm) were collected in series.

### Lesion Volume and Neural Degeneration

Coronal sections were collected at days 1, 4 and 10 post-stroke. Sections were stained with cresyl violet (Sigma-Aldrich) for lesion volume analysis. Lesion, defined as morphological changes in tissue, lack of staining, or pallor, was measured in a pre-calculated reference area using ImageJ software (National Institute of Health, Bethesda, MA, USA). The sections were imaged at 240 μm intervals and the lesion volume was calculated using the formula (lesion area) × (distance between sections).

To assess neural degeneration, Fluoro Jade (FJ) C staining was performed. A stock solution of FJC was prepared (25 mg FJC in 250 mL dH_2_O). Slides were immersed in 1% NaOH in 80% ethanol for 5 min, rinsed in 70% ethanol and distilled H_2_O for 2 min, each. Next, slides were in incubated in 0.06% KMnO_4_ for 10 min and then rinsed again in dH_2_O for 2 min. The following day, slides were incubated in a working solution of FJC (2 ml of stock solution in 198 mL dH_2_O and 198 mL acetic acid) for 30 min and washed in dH_2_O prior to cover slipping. The total numbers of FJC+ cells were manually counted using ImageJ (NIH) from every 10th section (i.e., 200 μm apart) between +2.3 and +1.10 mm from Bregma, within a pre-determined region of interest. Within a brain, coronal sections were divided into two clusters based on morphological characteristics (anterior and posterior to the genu of the corpus callosum). The total volume of the region of interest was 4.64 mm^3^, a sum of cluster 1 (2.55 × 1.0 × 1.6 mm) and cluster 2 (1.5 × 1.0 × 1.16 mm) volumes. The region of interest was kept consistent between all brains.

### Statistical Analysis

Statistical analyses were performed using Graphpad Prism v6.0 and Microsoft Excel 2011. Two-tailed *t*-tests were used for two group comparisons and two-way repeated measures ANOVAs were used for multiple group comparisons across more than one trial. For multiple group comparisons, *post hoc* tests Tukey or Dunnett were used when required based on group comparisons against each other, or group comparisons against one fixed control group, respectively. All data are reported as mean ± SEM. Statistical significance was considered when *p* ≤ 0.05.

## Results

### mPFC Stroke Leads to Targeted Lesions and Persistent Cognitive Impairments

**Figure 1:**
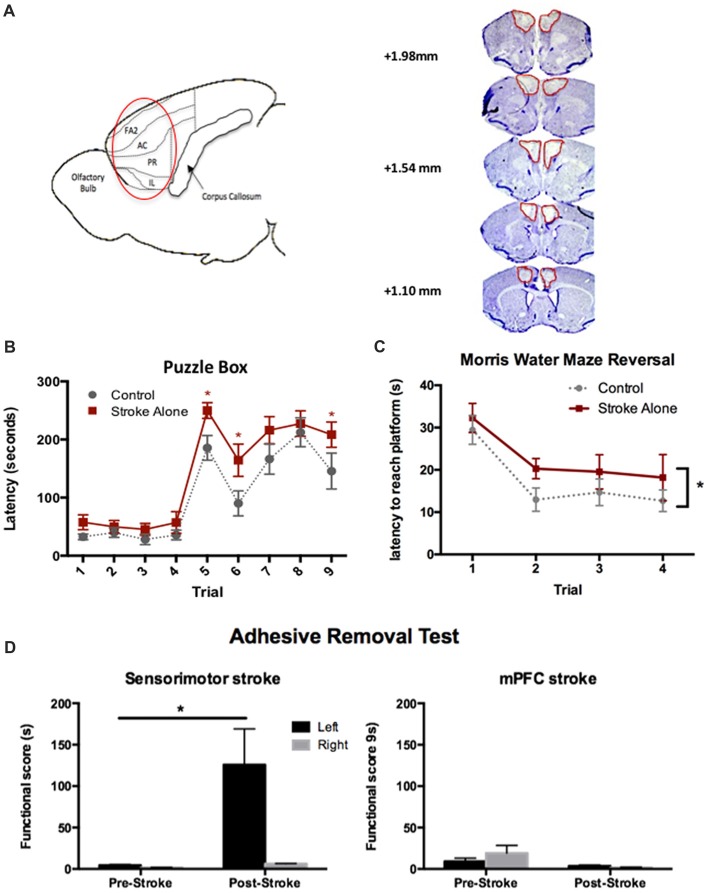
Endothelin-1 (ET-1)-induced cognitive model of stroke in mice. **(A)** Sagittal representation of the lesioned areas of the brain (circled). FA2, frontal area 2; AC, anterior cingulate cortex; PR, prelimbic; IL, infralimbic; cresyl violet stained coronal sections depicting typical medial prefrontal cortex (mPFC) lesion on day 4 post-stroke. **(B)** Significant cognitive impairments following stroke were detected using the Puzzle Box (PB) task at day 4–6 compared to control (*n* = 18 stroke; 16 control). **(C)** Following stroke, a significant cognitive deficit was detected up to 45 days, using the Morris Water Maze (MWM) reversal test (*n* = 12 stroke; 10 control). **(D)** Post-stroke functional scores in the adhesive removal test. Unilateral sensory-motor (*n* = 3), but not bilateral mPFC (*n* = 7) stroke, resulted in contralesional (left) forepaw deficits compared to baseline performance. *p < 0.05.

We first sought to establish a stroke model in mice that would result in persistent cognitive impairments using a bilateral injury model similar to those previously described in rats (Cordova et al., 2014; Livingston-Thomas et al., 2015). Bilateral ET-1 injections resulted in damage confined to the prelimbic, infralimbic and anterior cingulate subdivisions of the mPFC. Lesions spanned from approximately 2.34 mm to 1.10 mm anterior to Bregma, producing an average volume of 4.98 ± 0.92 mm^3^ at 4 days post-stroke (Figure 1A).

To assess cognitive outcome in the acute post-stroke phase, performance in the PB task was assessed at 4–6 days following injury. A cognitive deficit was defined as a significantly increased latency to enter the goal box compared to controls. In Stroke-alone mice, a significant deficit was detected on Trials 5, 6 and 9 (Stroke-alone: Trial 5: 249.94 ± 14 s; Trial 6: 164.39 ± 27 s; Trial 9: 208.39 ± 22 s; Controls: Trial 5: 185.67 ± 21 s; Trial 6: 90.13 ± 21 s; Trial 9: 145.67 ± 31 s;* p* < 0.05; Figure 1B). Therefore, bilateral mPFC stroke produced acute cognitive impairments in problem-solving and short-term memory after stroke.

Separate cohorts of mice were used to evaluate chronic cognitive impairment using the MWM test and Y-maze task. In the MWM test, there were no deficits detected in the acquisition or probe phases of the test (*p* > 0.05 for both); however, in the reversal phase, mPFC stroke animals exhibited a significant impairment, with a higher overall latency to find the platform compared to controls (Control: 17.5 ± 1.8 s; Stroke-alone: 22.6 ± 2.1 s; *F*_(1,20)_ = 4.751, *p* = 0.045; Figure 1C).

The Y-maze spontaneous alternation task was used to assess whether mPFC stroke resulted in deficits in frontal lobe-mediated spatial working memory on day 49 post-stroke. There were no significant differences in the spontaneous alternation percentage (SAP) between groups (data not shown). This demonstrates that mPFC stroke resulted in persistent chronic impairment in behavioral flexibility without affecting spatial working memory.

To rule out any confounding effect of sensorimotor impairments on cognitive tests, we used the adhesive removal test (Bouet et al., 2009). As predicted, mice with unilateral sensorimotor stroke lesions exhibited a significant contralateral (left) impairment (125.67 ± 43.44 s (left), 6.25 ± 0.39 s (right); *p* < 0.01; Figure 1D). Sensorimotor impairment was not observed in the mPFC lesioned mice (*p* > 0.05), consistent with histological examination of affected brain regions revealing no damage to the sensorimotor cortex.

### CsA Administration Expands the Size of the NPC Pool but Is Not Neuroprotective

**Figure 2:**
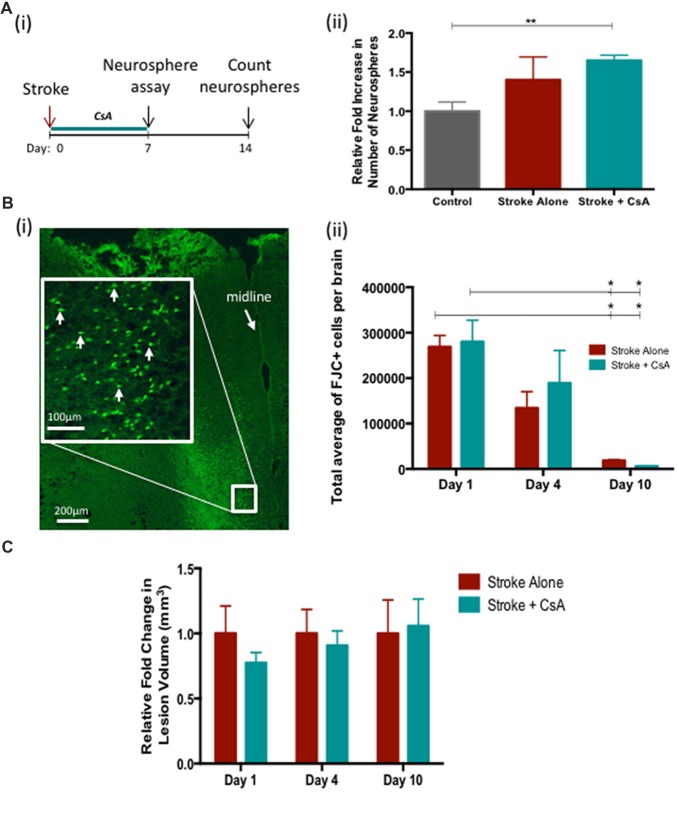
Cyclosporin A (CsA) expands the neural precursor cell (NPC) pool and is not neuroprotective. **(Ai)** Timeline of the experiment. Neurosphere assay was performed on day 7 following Stroke and counted 7 days later. **(ii)** Stroke-Alone (*n* = 6) results in a slight 1.4 fold increase, while Stroke+CsA (*n* = 5) caused a significant 1.7 fold increase (*n* = 7 controls); **p < 0.01. **(Bi)** Fluoro Jade C+ (FJC+) cells in the lesioned mPFC (white arrows). **(ii)** There was no significant difference in FJC+ cell counts between Stroke-Alone and Stroke+CsA groups on day 1 (*n* = 5/group), 4 (*n* = 5/group) or 10 (*n* = 3/group) post-stroke. **(C)** Lesion volumes are not significantly different between stroke-alone and stroke+CsA groups (*n* = 5/group) *p < 0.05.

Next we investigated whether administration of CsA from the time of stroke would have an effect on recovery. First, the neurosphere assay was performed on the SE at day 7 post-stroke to evaluate the effect of stroke and CsA treatment on the size of the NPC pool (Figure 2Ai). We observed a significant expansion in the size of the NPC pool in Stroke+CsA mice (1.7 ± 0.04 fold; Figure 2Aii). A slight non-significant expansion was observed in Stroke-alone mice (Control: 39 ± 8 neurospheres; Stroke-alone: 54 ± 2 neurospheres; Stroke+CsA: 65 ± 16 neurospheres per 5000 cells; *p* < 0.01). Thus, CsA treatment following mPFC stroke significantly expands the NPC population within the first week.

To determine if CsA delivery was neuroprotective post-stroke, the numbers of FJC+ cells and the lesion volume were analyzed on days 1, 4 and 10 after mPFC stroke. There were no significant differences in the number of FJC+ cells in Stroke-alone vs. Stroke+CsA groups at any of these time points (day 1 Stroke-alone: 2,68,799 ± 24,878 cells, Stroke+CsA: 2,80,089 ± 47,471 cells; day 4 Stroke-alone: 1,34,071 ± 36,015 cells; Stroke+CsA: 1,89,036 ± 71,746 cells; day 10 Stroke-alone: 18,480 ± 2372 cells; Stroke+CsA: 6209 ± 655 cells; Figures 2Bi,ii). In both the Stroke-alone and Stroke+CsA groups, the number of FJC+ cells declined from day 1 to 10 post-stroke (*p* < 0.05). There were no significant differences in lesion volume between the Stroke-alone and Stroke+CsA groups at any time point (Figure 2C). Taken together, these findings demonstrate that CsA treatment did not have neuroprotective effects when administration was started immediately after stroke.

### Chronic CsA Administration Rescues Cognitive Impairment Following mPFC Stroke in the Puzzle Box Task

**Figure 3:**
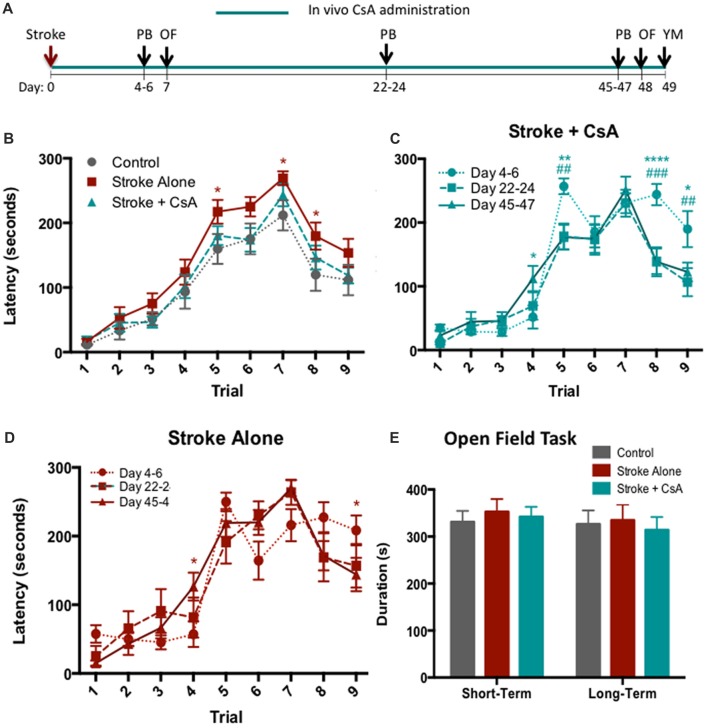
Chronic CsA treatment reduces cognitive impairments.** (A)** Experimental timeline. PB, puzzle box; OF, Open Field; YM, Y-Maze. **(B)** Stroke-Alone (*n* = 18), but not Stroke+CsA (*n* = 16), showed persisting cognitive deficits compared to controls (*n* = 14) in the PB task; *p < 0.05. **(C)** Stroke+CsA animals show significant improvement at day 22–24 (#) and 45–47 (*) compared to day 4–6. **(D)** Stroke-alone animals show spontaneous recovery in trial 9 at long-term time points. **(E)** No significant differences were observed in the amount of time spent in the corner zones of the maze between Stroke-alone (*n* = 11), Stroke+CsA (*n* = 11), and control (*n* = 8) groups. Day 22–24: *p < 0.05, **p < 0.01, ****p < 0.0001; Day 45–47: ^#^*p* < 0.05, ^##^*p* < 0.01, ^###^*p* < 0.001.

Cognitive performance following CsA treatment was assessed at early (post-stroke days 4–6), middle (post-stroke days 22–24), and late (post-stroke days 45–47) time points using the PB (Figure 3A). Early post-stroke deficits were not improved in the CsA-treated mice. However, at both middle and late time points, Stroke+CsA animals were recovered to control level performance, while Stroke-alone animals continued to exhibit deficits (Figure 3B). Late time point data were combined, revealing that overall, the Stroke-alone mice exhibited significant impairments on Trials 5 (Stroke-alone: 217.25 ± 18 s; Control: 159.79 ± 23 s; *p* < 0.05), Trial 7 (Stroke-alone: 269.11 ± 11 s; Control: 211.82 ± 23 s; *p* < 0.05) and Trial 8 (Stroke-alone: 179.67 ± 21 s; Control: 119.79 ± 25 s; *p* < 0.05), whereas the Stroke+CsA group had recovered to Control performance (Trial 5: 180.44 ± 15 s; Trial 7: 243.19 ± 17 s; Trial 8: 146.38 ± 19 s). Hence, CsA administration reduced long-term cognitive deficits.

We further compared the PB performance in Stroke-alone and Stroke+CsA mice at each of the time points tested. At day 22–24, Stroke+CsA mice showed reduced latency to reach the goal box on Trials 5 and 8 (Trial 5: 178.50 ± 20 s; Trial 8: 139.42 ± 20 s) compared to performance on day 4–6 (Trial 5: 256.94 ± 12 s; Trial 8: 244.00 ± 17 s; *p* < 0.01, *p* < 0.001), suggesting an improvement in the problem-solving deficit that persisted until day 45–47 (Trial 5: 177.13 ± 20 s; Trial 8: 138.56 ± 23 s; Figure 3C). The Stroke-alone group did not show any significant changes in performance on Trials 5 and 8 at any of the long-term test points (Figure 3D). These findings further support the hypothesis that long-term CsA treatment reduces cognitive impairments following stroke.

To confirm that the increased latency for stroke animals to complete trials in the PB task was not confounded by alterations in anxiety, the open field task was performed at early (day 7) and late (day 48) time points post-stroke (Figure 3A). No significant differences were observed in the amount of time spent in corner zones in any of the groups at either time point (Figure 3E). Similarly, the total distance traveled in the corner zones was not significantly different (data not shown). Hence, locomotion and anxiety were not affected by mPFC stroke.

### NPCS Migrate to the Site of Injury Following mPFC Stroke

Previous findings have shown NPC migration to the site of the injury following sensorimotor stroke, in the presence and absence of CsA treatment (Sachewsky et al., 2014; Faiz et al., 2015). We investigated whether similar NPC migration was seen in this mPFC stroke model. On day 60 post-stroke, neurospheres were observed in the cortex of the stroke injured mice, but not controls (Stroke-alone and Stroke+CsA cortical neurospheres in 14% and 43% of brains, respectively, compared to 0% of brains in controls). Hence, mPFC stroke can promote NPC migration toward the site of injury, and this migration can be enhanced following CsA administration.

## Discussion

In this study, we investigated the efficacy of endogenous NPC activation using CsA to promote cognitive recovery following mPFC stroke. We successfully reproduced a cognitive stroke model in mice. Using the PB and MWM tasks, we demonstrated that mPFC injury resulted in cognitive deficits across various cognitive domains, detectable as early as 4 days and persisting up to 45 days post-stroke, similar to previous findings in rats (Cordova et al., 2014; Livingston-Thomas et al., 2015). Short-term CsA treatment expanded the SE-derived NPC pool early after stroke, and chronic post-stroke CsA administration promoted migration of NPCs and recovery of persistent long-term cognitive deficits. Hence, we have demonstrated that an endogenous NPC activation strategy using the immunosuppressant CsA has the potential to enhance recovery following mPFC stroke.

Sensorimotor stroke results in the transient expansion of the SE NPC pool 7 days post-injury, a phenomenon not observed in the present study. It is possible that the injury induced expansion of the NPC pool in this model at an earlier time post-stroke. Alternatively, the lack of effect observed in this model may be due to the location of the injury; the mPFC is more distant from the SE than the sensorimotor cortex. This finding could have implications for the translation of NPC migration approaches as the degree of NPC activation would vary depending on the site of the stroke lesion. Nonetheless, post-stroke administration of CsA led to a significant expansion of the NPC pool, similar to the effect seen following CsA administration in sensorimotor stroke (Sachewsky et al., 2014).

Our findings reveal that despite CsA’s well-documented neuroprotective effects (Sullivan et al., 2011; Yousuf et al., 2011), delivery of CsA did not result in sparing of tissue or neuroprotection post-stroke. Our data suggest that activation of endogenous precursors is sufficient for promoting recovery post-stroke. This is consistent with our previous work showing that a CsA analog, NIM811, is able to promote sensorimotor functional recovery following cortical stroke despite its non-immunosuppressive effects (Sachewsky et al., 2014). Notably, our current findings do not eliminate a potential role for suppressing neuroinflammation in cognitive recovery. To test whether NPC activation is sufficient for recovery as suggested herein, and not due in part to immunosuppression, it would be interesting to explore the effects of NIM811 in this model.

The development of this reproducible model of mPFC stroke provides a foundation to examine the efficacy of novel therapeutics to enhance recovery. Here, we showed that following injury, administration of the immunosuppressant drug CsA can activate NPCs in the adult forebrain and reduce cognitive impairments. Investigating the efficacy of combinatorial strategies (i.e., rehabilitation) alongside small molecule activation strategies to accelerate post-stroke cognitive recovery, as previously shown in sensorimotor stroke (Jeffers et al., 2014), would be a logical next step toward advancing knowledge in this area.

## Author Contributions

LN was responsible for performing surgeries, experiments, data analyses and interpretation and writing of the manuscript. JML-T conducted the MWM task to assess chronic cognitive impairments and contributed in writing the manuscript. VR was responsible for lesion volume and neural degeneration analyses. KA assisted in performing the neurosphere assays. IV assisted in conducting adhesive tape removal test and subsequent data analyses. DC was involved in providing technical and academic guidance on the project and editing of the manuscript. CMM was the principal investigator of this project and was involved in writing of the manuscript.

## Conflict of Interest Statement

The authors declare that the research was conducted in the absence of any commercial or financial relationships that could be construed as a potential conflict of interest.
